# Data on hematological parameters and generalized severe periodontitis in the United States

**DOI:** 10.1016/j.dib.2023.110010

**Published:** 2024-01-05

**Authors:** Menglin Hu, Wenhao Zhang, Zhimin Shi, Xiaoyun Liu, Xu Cheng, Hengguo Zhang, Yuanyin Wang

**Affiliations:** a Key Laboratory of Oral Diseases Research of Anhui Province, College & Hospital of Stomatology, Anhui Medical University, Hefei 230032, China; b Department of Dental, Tongling Traditional Chinese Medicine Hospital, Taipinghu Road, Tongling 244000, China; c Department of Dental Implantology, College & Hospital of Stomatology, Anhui Medical University, Hefei 230032, China; d Department of Oral and Maxillofacial Surgery, College & Hospital of Stomatology, Anhui Medical University, Hefei 230032, China

**Keywords:** Periodontitis, Public health, Statistics, Retrospective study

## Abstract

This article describes an ensemble of datasets used to understand the relationship between generalized severe periodontitis and hematological parameters. This dataset combines public periodontal examination data, hematological parameters data, and demographic data from the National Center for Health Statistics from 2009 to 2014. The stage of periodontitis was identified by attachment loss conducted by dental examiners, who were dentists (D.D.S./ D.M.D.) licensed in at least one U.S. state, while matching current classification criteria from the American Academy of Periodontology and the European Federation of Periodontology. Based on the NHANES database, information on age, gender, education level (< 9th grade, 9–11th grade, high school, college, graduate), race/ethnicity (Mexican American, Hispanic, non-Hispanic White, non-Hispanic Black, and other races), PIR (poverty income ratio) were acquired from the demographic data. Hematological parameters (including HB, HCT, mean cell volume, mean cell hemoglobin, mean cell hemoglobin concentration, red cell distribution width, platelet count, mean platelet volume, and red blood cell count) and glucose data had been obtained from laboratory data. Smoking data had been obtained from questionnaire data.

Specifications Table

**Table:** 

Subject	Dentistry, Oral Surgery and Medicine
Specific subject area	Health and medical sciences
Data format	This is a secondary analysis of data from the NHANES 2009–2014 survey.
Type of data	Table
Data collection	NHANES 2009–2014 Data Files https://wwwn.cdc.gov/nchs/nhanes/Default.aspx
Data source location	Primary Data Sources: NHANES 2009–2014 Data Files, Examination Files, Laboratory Files, Demographics Files, Questionnaire Data Country: USA NIS: https://www.cdc.gov/nchs/nhanes/
Data accessibility	Repository name: hematological parameters and generalized severe periodontitis Direct URL to data: https://wwwn.cdc.gov/nchs/nhanes/search/datapage.aspx?Component=Demographics&Cycle=2013-2014 https://wwwn.cdc.gov/nchs/nhanes/search/datapage.aspx?Component=Demographics&Cycle=2011-2012 https://wwwn.cdc.gov/nchs/nhanes/search/datapage.aspx?Component=Demographics&Cycle=2011-2012 https://wwwn.cdc.gov/nchs/nhanes/search/datapage.aspx?Component=Examination&Cycle=2013-2014 https://wwwn.cdc.gov/nchs/nhanes/search/datapage.aspx?Component=Examination&Cycle=2011-2012 https://wwwn.cdc.gov/nchs/nhanes/search/datapage.aspx?Component=Examination&Cycle=2009-2010 https://wwwn.cdc.gov/nchs/nhanes/search/datapage.aspx?Component=Laboratory&Cycle=2013-2014 https://wwwn.cdc.gov/nchs/nhanes/search/datapage.aspx?Component=Laboratory&Cycle=2011-2012 https://wwwn.cdc.gov/nchs/nhanes/search/datapage.aspx?Component=Laboratory&Cycle=2009-2010 https://wwwn.cdc.gov/nchs/nhanes/search/datapage.aspx?Component=Questionnaire&Cycle=2013-2014 https://wwwn.cdc.gov/nchs/nhanes/search/datapage.aspx?Component=Questionnaire&Cycle=2011-2012 https://wwwn.cdc.gov/nchs/nhanes/search/datapage.aspx?Component=Questionnaire&Cycle=2009-2010 Instructions for accessing these data: Raw data can be retrieved from NHANES 2009-2014 Series 11 Data Files
Related research article	Zhang W, Zhang Y, Jin C, et al. The indicative role of inflammatory index in the progression of periodontal attachment loss. Eur J Med Res. 2023;28(1):287. Published 2023 Aug 17. doi:10.1186/s40001-023-01247-8

## 1 Value of the Data

• Compared with similar datasets, this dataset provides data on hematological parameters in patients with periodontitis stages, which reflect changes in the progression of periodontitis symptoms.
• These data can be of benefit to researchers who are interested in factors associated with periodontitis progression research.
• These data will also allow clinicians and researchers to compare hematological parameters, behavioral and demographic disparities in patients with different levels of periodontitis.

## 2 Data Description

This dataset contains information from NHANES on periodontal examination outcomes for 5396 U.S. patients between 2009 and 2014. Based on the current classification of periodontitis in 2018, 5396 participants were divided into three groups (stage I periodontitis, stage II periodontitis, stage III and IV periodontitis) based on attachment loss and missing teeth [1]. The dataset also includes demographic data (age, gender, race, person education level, PIR), laboratory data (white blood cell count, lymphocyte percent, monocyte percent, segmented neutrophils percent, eosinophils percent, basophils percent, lymphocyte number, monocyte number, segmented neutrophils num, eosinophils number, basophils number, red blood cell count, hemoglobin, mean cell volume, mean cell hemoglobin, mean cell hemoglobin concentration, red cell distribution width, platelet count, mean platelet volume, plasma fasting glucose) and questionnaire data (cigarette use). The raw data files were deposited in the NHANES data repository https://www.cdc.gov/nchs/nhanes/. The data information in the sample is shown in Table 1.

**Table 1:** Characteristics of the study populations according to periodontitis status in the US young adult population (NHANES, 2009–2014).

	Stage I periodontitis	Stage II periodontitis	Stage III and IV periodontitis
Participants (n)	316	842	4238
Age (n)	39.71±9.06	43.50±11.29	56.97±13.53
Gender (n%)			
male	112[35.44]	411[48.81]	2518[59.41]
female	204[64.56]	431[51.19]	1720[40.58]
Race (n%)			
Mexican American	22[6.96]	112[13.30]	726[17.13]
Other Hispanic	23[7.28]	72[8.55]	443[10.45]
Non-Hispanic White	187[59.18]	442[52.49]	1554[36.67]
Non-Hispanic Black	42[13.29]	113[13.42]	1049[24.75]
Other Race - Including Multi-Racial	42[13.29]	103[12.23]	466[11.00]
Person Education Level (n%)			
Less Than 9th Grade	8[2.53]	44[5.23]	630[14.87]
9–11th Grade	14[4.43]	47[5.58]	734[17.32]
High School Grad/GED or equivalent	48[15.19]	103[12.23]	1033[24.37]
Some College or AA degree	99[31.33]	230[27.32]	1067[25.18]
College Graduate or above	147[46.52]	418[49.64]	766[18.07]
Refused	0[0]	0[0]	3[0.07]
Don't Know	0[0]	0[0]	5[0.12]
PIR	3.46±1.57	3.32±1.63	2.31±1.57
WBC (1000 cells/uL)	6.87±2.01	6.93±2.05	7.22±2.90
LY percent (%)	31.61±7.97	30.78±8.03	30.29±8.99
MONO percent (%)	7.43±1.95	7.65±2.31	7.86±2.63
NE percent (%)	57.64±9.02	58.19±8.87	58.24±10.03
EOS percent (%)	2.64±1.63	2.72±2.07	2.95±2.19
BASO percent (%)	0.73±0.47	0.69±0.51	0.71±0.58
LY (1000 cells/uL)	2.11±0.62	2.07±0.66	2.13±1.15
MONO (1000 cells/uL)	0.49±0.16	0.51±0.17	0.55±0.21
NEUT (1000 cells/uL)	4.04±1.68	4.11±1.64	4.29±2.31
EOS (1000 cells/uL)	0.18±0.11	0.19±0.19	0.21±0.22
BASO (1000 cells/uL)	0.04±0.05	0.04±0.06	0.04±0.10
RBC (million cells/uL)	4.57±0.46	4.63±0.46	4.60±0.51
HB (g / dL)	13.94±1.37	14.17±1.43	14.07±1.50
HCT (%)	40.62±3.81	41.37±4.03	41.33±4.27
MCV (fL)	89.05±5.34	89.56±4.98	90.06±5.97
MCH (pg)	30.55±2.16	30.67±2.03	30.68±2.49
MCHC(g/dL)	34.30±1.02	34.23±0.99	34.04±1.14
RDW(%)	12.78±1.02	12.94±1.28	13.21±1.34
PLT (1000 cells/uL)	244.02±57.44	236.22±54.69	234.65±64.95
MPV (fL)	8.20±0.98	8.20±0.92	8.17±0.96
GLU(mmol/L)	5.40±1.06	5.70±1.34	6.33±2.13
NA	165	427	2288
Smoked at least 100 cigarettes in life			
yes	89[28.16]	288[34.20]	2225[52.50]
no	227[71.84]	554[65.80]	2012[47.48]
Refused	0[0]	0[0]	1[0.02]
Age started smoking			
Never	7[2.22]	19[2.26]	70[1.65]
age	17.95±4.15[25.95]	17.97±4.10[31.95]	18.31±5.72[50.66]
NA	227[71.84]	554[67.80]	2021[47.69]
Do you now smoke cigarettes			
Every day	23[7.28]	81[9.62]	861[20.32]
Some days	7[2.22]	31[3.68]	158[3.73]
Not at all	59[18.67]	176[20.90]	1205[28.43]
NA	227[71.84]	554[65.79]	2014[47.52]
Quit smoking time			
years	3.68±1.06[18.67]	14.89±12.29[20.90]	19.84±14.35[28.08]
NA	257[81.33]	666[79.10]	3048[71.92]
Number of smokers in the household			
0	6[1.90]	26[3.09]	75[1.77]
1	8[2.53]	27[3.21]	399[9.414]
2	11[3.48]	18[2.14]	248[5.85]
3	0[0]	2[0.24]	58[1.37]
NA	291[92.09]	769[91.33]	3458[81.60]

## 3 Experimental Design, Materials and Methods

Periodontal exam data, hematological parameters, demographic and smoking data were collected from the CDC National Survey, which can be downloaded from the CDC website.

Periodontal, hematological parameters and cigarette use data were from the National Health and Nutrition Examination Surveys (NHANES), which can be downloaded from the Centers for Disease Control and Prevention (CDC) official website. The NHANES is a program of studies designed to assess the health and nutritional status of adults and children in the United States. The survey used a combination of interviews and physical examinations to gather information about the participants.

We processed the raw data in two significant ways. First, we obtained preliminary stage classification of periodontitis in participants by labeling the degree of attachment loss in each tooth. In the classification of stage I periodontitis, the attachment loss of teeth was 1 to 2 mm and there was no tooth loss caused by periodontitis. In the classification of stage II periodontitis, the attachment loss of teeth was 3 to 4 mm and there was no tooth loss caused by periodontitis. The patients with tooth attachment loss of 5 mm or more were classified as stage III and IV periodontitis [2]. Second, we excluded patients with missing teeth from the stage I and II periodontitis classification to obtain the final stage classification of periodontitis. The questionnaire data, demographic data, and laboratory data were included in the table according to the final classification.

The periodontal data obtained were analyzed and presented using descriptive statistics such as analysis of variance. In Table 1, the continuous variables were presented as the mean (standard deviation) and the categorical variables were presented as the number (percentage) [3], [4], [5], [6].

## Limitations

It was not possible to determine whether the loss of teeth in the dataset was caused by periodontitis. Therefore, when counting the number of patients with stage I and stage II periodontitis, we excluded patients with missing teeth, which resulted in a smaller sample size of patients with stage I and stage II periodontitis.

## Ethics Statement

The studies involving human participants were approved by the institutional review board of the National Center for Health Statistics, CDC, and were conducted in accordance with the Helsinki Declaration of 1975, as revised in 2013. The patients/participants provided their written informed consent to participate in this study.

## CRediT authorship contribution statement

**Menglin Hu:** Conceptualization, Methodology, Software, Validation, Investigation, Data curation, Writing – original draft, Writing – review & editing. **Wenhao Zhang:** Conceptualization, Methodology. **Zhimin Shi:** Investigation, Data curation, Writing – review & editing. **Xiaoyun Liu:** Methodology, Software. **Xu Cheng:** Validation, Investigation, Writing – original draft. **Hengguo Zhang:** Conceptualization, Methodology, Investigation, Resources, Writing – review & editing, Supervision, Project administration, Funding acquisition. **Yuanyin Wang:** Conceptualization, Resources, Writing – review & editing, Supervision, Project administration.

## Data Availability

National Health and Nutrition Examination Survey (Reference data) (Centers for Disease Control and Prevention)
